# Epigenetic dysregulation of immune-related pathways in cancer: bioinformatics tools and visualization

**DOI:** 10.1038/s12276-021-00612-z

**Published:** 2021-05-07

**Authors:** Anders Berglund, Ryan M. Putney, Imene Hamaidi, Sungjune Kim

**Affiliations:** 1 Departments of Biostatistics and Bioinformatics, H. Lee Moffitt Cancer Center & Research Institute, Tampa, FL USA; 2 Department of Radiation Oncology, H. Lee Moffitt Cancer Center & Research Institute, Tampa, FL USA

**Keywords:** Genetics research, Biomarkers

## Abstract

Cancer immune evasion is one of the hallmarks of carcinogenesis. Cancer cells employ multiple mechanisms to avoid immune recognition and suppress antitumor immune responses. Recently, accumulating evidence has indicated that immune-related pathways are epigenetically dysregulated in cancer. Most importantly, the epigenetic footprint of immune-related pathways is associated with the patient outcome, underscoring the crucial need to understand this process. In this review, we summarize the current evidence for epigenetic regulation of immune-related pathways in cancer and describe bioinformatics tools, informative visualization techniques, and resources to help decipher the cancer epigenome.

## Cancer: Bioinformatics helps identify immune system evasion

Abnormal patterns of genomic chemical modification help tumors elude immunological destruction, but sophisticated computational tools could help identify and overcome these survival mechanisms. Immunotherapy can be a potent weapon against cancer, but many tumors evolve the ability to protect themselves by subduing the immune response. Sungjune Kim and colleagues at the Moffitt Cancer Center, Tampa, USA, have reviewed efforts to study how chemical alterations to DNA that affect gene expression contribute to this process. Considerable evidence indicates a role for a modification called methylation in this immune evasion, and researchers now have access to vast repositories of tumor-specific gene methylation profiles. The authors describe these data resources, and highlight some of the software tools that are helping oncologists to identify patterns in the data that might lead to better therapies.

## Introduction

Cancer immunotherapy constitutes a major paradigm shift in cancer care. Recent successes with immune checkpoint inhibitors, most notably CTLA4^1,2^ and PD1/PDL-1^3–6^ inhibitors, have altered the landscape of systemic therapy for cancer. Despite such breakthroughs, the majority of cancer patients remain refractory to existing cancer immunotherapeutic modalities, highlighting the inherent capacity of tumor cells to evade the immune system. Mechanisms that impede immune surveillance during carcinogenesis are required for tumor cells to progress towards the development of macroscopic tumors. Indeed, cancer cells regulate immune-related pathways to suppress the immune system^7^ via intricate modulation at the transcriptional, translational, and posttranslational levels^7–9^.

Echoing the pivotal importance of cancer immune evasion, immune-related pathways are frequently dysregulated in cancer. However, for heritable changes to impact the entire tumor tissue, the initial cascade of tolerogenic signals must involve genetic or epigenetic changes, and accordingly, the mutational landscape of cancer includes crucial immune pathways regulating tumor immunity in a subset of patients^7^. However, loss or gain of function via somatic mutations are rare events, whereas every tumor is required to acquire a tolerogenic immune barrier to survive. In this context, there is a growing consensus that the immune-evasive phenotype of cancer cells relies in part on the epigenetic machinery, which is based on changes that in turn make cancer cells more adaptable. Indeed, cancer cells frequently utilize epigenetic dysregulation to silence tumor suppressors or activate oncogenes^10^; similarly, carcinogenesis may also require epigenetic reprogramming of immune-related pathways to evade immune killing.

Methylation is one of the major epigenetic mechanisms modulating gene transcription in cancer. Furthermore, increased stochastic variations in methylation events are manifested in the cancer epigenome, thus contributing to tumor heterogeneity^11^. Recent studies have also demonstrated the crucial role of dysregulated methylation in modulating tumor immunity^12^. Therefore, in this review, we outline the key immune pathways active in cancer cells, the relevance of methylation in regulating these pathways, and the bioinformatic methodologies and resources to decipher the epigenetic manifestations of cancer immune evasion.

## The immune synapse

**Fig. 1:**
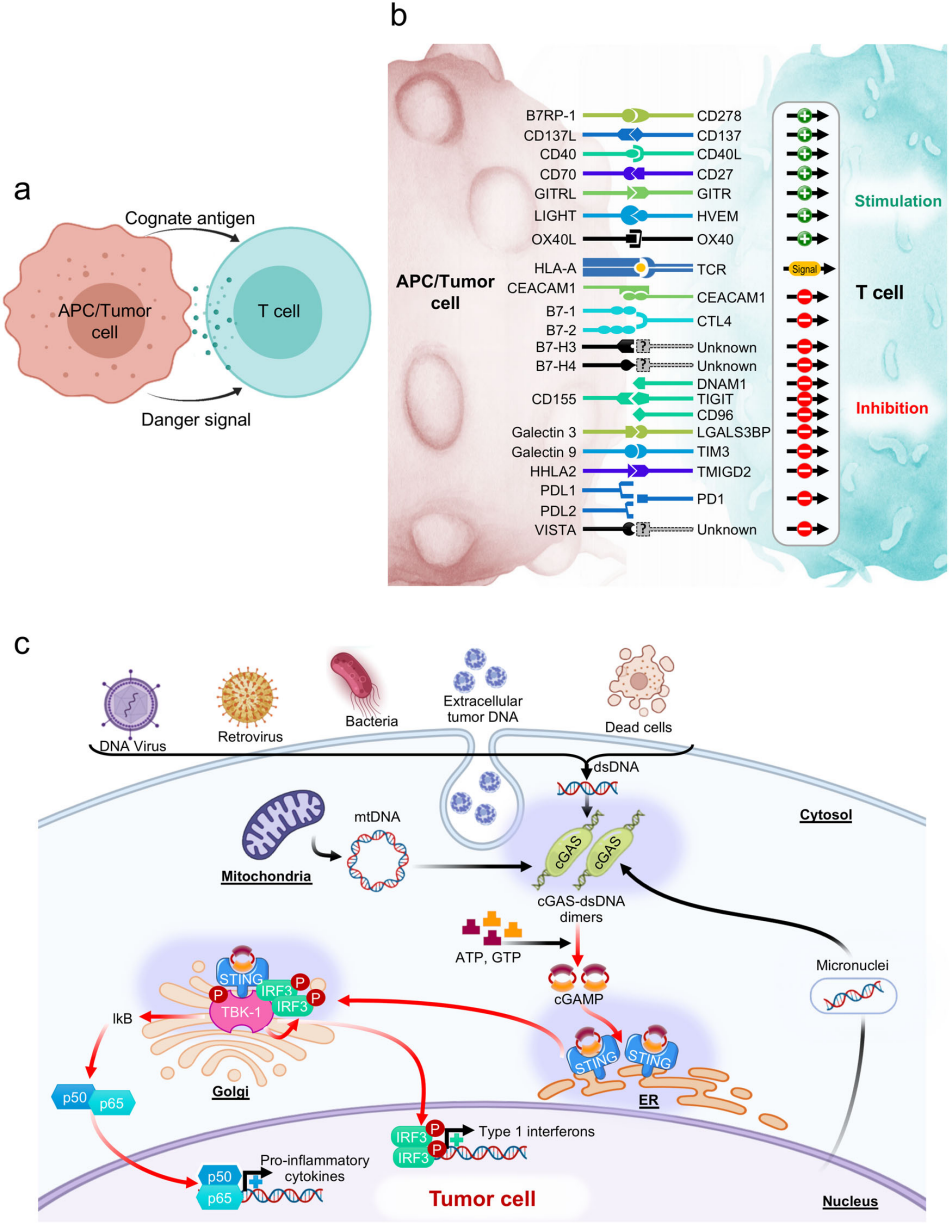
Immune-related pathways in cancer. **a** Schematic of the prototypic immune synapse. **b** Expanded depiction of the immune synapse between APCs/tumor cells and T cells. A comprehensive interaction between costimulatory and immune checkpoint ligand-receptor pairs is visualized. **c** STING-cGAS signaling pathway. Sensing of double-stranded DNA by cGAS leads to endogenous generation of cGAMP, which stimulates STING tetramerization and downstream signaling through IRF3, TBK1, and NF-κB to elicit interferon signaling.

The immune synapse between Antigen-presenting cells (APCs) and cognate T cells relays two signals. Signal 1 refers to the presentation of a specific peptide antigen by major histocompatibility complex (MHC) molecules on APCs to the cognate T cell receptor on tumor-specific T cells. Signal 2 is the “danger signal” that alerts the immune system to elicit functional immune responses via engagement of costimulatory molecules, degradation of immune checkpoints, and release of proinflammatory cytokines, as APCs recognize conserved danger motifs, such as Toll-like receptor ligands, pathogen-associated molecular patterns (PAMPs), or damage-associated molecular patterns (DAMPs). Indeed, the prototypic immune synapse (Fig. 1a) has now been expanded to encompass the complex interaction between APCs and effector T cells (Fig. 1b), and the qualitative and quantitative signal transmitted through the immune synapse determines the ensuing antitumor immune response. Consistent with this observation, many of the molecules comprising the immune synapse, including but not limited to PD1, CTLA4, Tim3, LAG3, and TIGIT, are targeted by the current generation of cancer immunotherapy and new pipeline drugs (Fig. 1b).

More recently, STING and cyclic GMP-AMP synthase (cGAS) have gained considerable attention as representing the pivotal pathway that regulates downstream interferon signaling to provide danger signals. The abnormal presence of cytosolic DNA from dying tumor cells leads to cGAS activation and the generation of cyclic GMP-AMP, which, upon binding to STING, activates TANK-binding kinase 1 (TBK1) and IRF3, ultimately triggering type 1 interferon signaling^13^. The STING-cGAS pathway also induces NF-κB signaling and the transcription of proinflammatory cytokines, including IL-6 and TNFα (Fig. 1c)^13^.

## Methylation in cancer

DNA methylation of cytosine residues, primarily on 5’-C-phosphate-G-3 (CpG) dinucleotides, and covalent modifications of histones via acetylation, methylation, phosphorylation, and ubiquitination, confer a heritable epigenetic code that profoundly regulates transcriptional activity in normal tissues and cancer cells. Accumulating evidence shows that aberrant epigenetic reprogramming significantly contributes to tumor initiation and progression^14,15^. For instance, site-specific promoter hypermethylation of tumor suppressors has been considered the key epigenetic event during carcinogenesis^14^. More recent studies have shown increased stochastic variations in methylation events in the cancer epigenome^11^, which may contribute to tumor heterogeneity. Mechanistically, DNA methyltransferase 1 (DNMT1), DNMT3A, and DNMT3B have been shown to be important maintenance and de novo methyltransferases in cancer^16,17^. By contrast, ten-eleven translocation methylcytosine dioxygenase 1 (TET1), TET2 and TET3 mediate key steps in active DNA demethylation^18^.

## Dysregulated methylation of immune pathways in cancer

In recent years, it has become apparent that immune-related pathways in cancer are regulated by the epigenetic machinery. For instance, mechanisms of human leukocyte antigen (HLA) downregulation include mutations in MHC class I heavy chain genes^19,20^, mutations in the b2m gene^21^, mutations in genes encoding JAK/STAT pathway components^22^ and hypermethylation of MHC class I gene loci^23^. Furthermore, the expression of costimulatory and immune checkpoint molecules is not limited to immune cells; tumor cells also exploit these interactions to induce a tolerogenic tumor microenvironment^7^. Indeed, comprehensive profiling of the DNA methylation status of immune synapse genes using level 1 methylation data from 8,186 solid tumors and 745 normal adjacent tissues in 30 solid tumor types from The Cancer Genome Atlas (TCGA) demonstrated hypomethylation of immune checkpoint genes and hypermethylation of costimulatory genes across disease sites in comparison to normal adjacent tissues, thus indicating that these cells adopted an immune-tolerogenic phenotype^12^. Strikingly, the differential methylation status of the immune synapse genes exhibited prognostic significance in immunogenic cancers, including melanoma, lung cancer, kidney cancer, head and neck cancer, and breast cancer, with associated changes in effector T cell trafficking to the tumor microenvironment^12^.

Interestingly, the STING-cGAS pathway also appears to be epigenetically regulated in cancer cells^24^. The methylation status of the STING and cGAS genes in 8426 primary solid tumors and 747 normal adjacent tissues with data deposited in the TCGA database revealed relative hypermethylation of these crucial pathways to suppress cytosolic DNA sensing and interferon signaling^24^.

## Clinical relevance

As dysregulated methylation is deemed critical in modulating antitumor immune responses, translational studies to harness the immunogenic effects of manipulating tumor methylation are underway, with strong evidence from previous preclinical studies demonstrating the efficacy of demethylating agents to augment immunotherapy^25,26^. Importantly, recent success with demethylating agents in combination with anti-PD1 therapy (nivolumab) in AML suggests the clinical merit of this approach^27^. However, the negative preliminary findings from the phase II randomized clinical trial of oral 5-azacitidine plus pembrolizumab (a PD1 blocker) vs. pembrolizumab plus placebo in non-small-cell lung carcinoma^28^ suggest that patient selection may be crucial to harness the therapeutic efficacy of 5-azacitidine. Indeed, given the multifaceted immune tolerance mechanisms employed by cancer cells, the immunogenic effects of 5-azacitidine may be largely limited to patients with unfavorable methylation patterns, suggesting a need for biomarker development to optimize the use of demethylating agents to augment cancer immunotherapy in the clinic.

## The Illumina methylation array and bioinformatic pipelines for analysis

The Illumina HumanMethylation450 BeadChip (HM450) proved to be a success, providing an affordable, user-friendly, and high-throughput platform for measuring the DNA methylation status at single-base resolution^64^. HM450 evaluates over 480,000 CpG sites that predominantly targeting RefSeq genes and CpG islands with 99% and 96% coverage, respectively^65^.

The MethylationEPIC BeadChip (EPIC) utilizes the same technology and design principles as HM450, but the EPIC chip contains probes for over 850,000 CpG sites, retaining 91.1% percent of the probes on HM450^66^. EPIC expands coverage of the genome by including 413,745 CpG sites that were not on HM450, 333,265 of which newly target enhancer regions distal to transcription start sites^64,66^.

Due to the success and popularity of Illumina methylation BeadChips, there is a need for bioinformatics tools that are able to import, preprocess, visualize, and analyze the data produced by these platforms. Specialized software packages that target a specific step in the analysis pipeline are developed and released, often accompanying published research. For example, the implementation of a novel preprocessing method might be made freely available. For many researchers, however, comprehensive software packages that allow the user to easily perform the majority of the steps needed to convert raw data into results are of interest. Table 1 gives an overview of such packages. The majority of the packages are designed for the R programming language and environment^67^ and are available through the Bioconductor project^68,69^. Included are three alternatives: a Java-based package, a suite of Python modules, and a web-based application.

**Table 1:** Bioinformatic tools and pipelines for analyzing methylation data.

Name	Platfor	GUI	Data Types	QC	Preprocess	Cell	DMP	DMR
RnBeads^29,30^	R/Bioc	Yes	IDAT, betas, GEO, Bis-Seq bed	Control Plots, PCA/MDS	BMIQ^31^, SWAN^32^, dasen^33^, NOOB^34^	Yes	limma^35,36^, t-test, RefFreeEWAS^37^, rank-based	Aggregate *p*-value
ChAMP^38,39^	R/Bioc	No	IDAT, betas	Control Plots, PCA/MDS	BMIQ, FunNorm^40^, SWAN	Yes	limma	Probe Lasso^41^, bumphunter^42^, DMRcate^43^
SeSAMe^44^	R/Bioc	No	IDAT	QC statistics	Nonlinear dye^44^, NOOB	Yes	No	No
Minfi^45,46^	R/Bioc	No	IDAT, GEO, betas	Control Plots, Beta Density, MDS	SWAN, NOOB, FunNorm, SQN^47^, Illumina	Yes	limma	bumphunter
shinyÉPICo^48^	R/Bioc	Yes	IDAT	Beta Density, PCA	As minfi	No	limma	mCSEATest^49^
ENmix^50^	R/Bioc	No	IDAT	Control Plots, PCR	Enmix^51^, RELIC^52^, RCP^53^	Yes	No	comb-p^54^, ipDMR^55^
MADA^56^	Web	Yes	IDAT	Beta Density, MDS	As minfi, BMIQ, dasen	No	limma, samr^57^	Probe Lasso, bumphunter, DMRcate, seqlm^58^
FOXO BioScience^59–61^	Python	No	IDAT, GEO, ArrayExpress	Control Plots, Beta Density, MDS	NOOB	No	linear regression, logistic regression	No
DimMer^62,63^	Java	Yes	IDAT	No	Illumina, SQN	Yes	*t*-test, linear regression	sliding window

*Bioc* bioconductor, *MDS* multidimensional scaling, *PCA* principal component analysis, *PCR* principal component regression, *DMP* differentially methylated position, *DMR* differentially methylated region.

### Sources of methylation data

Many methylation datasets for different tumor types exist, many of which also include normal samples. Clinical data are frequently included, and some also include matching gene expression data. Below, we describe some of the key sources of methylation datasets, focused on methylation data based on the Illumina Infinium HumanMethylation27 BeadChip (HM27), Illumina Infinium HumanMethylation450 BeadChip (HM450), and Illumina Infinium MethylationEPIC BeadChip (EPIC).

The Cancer Genome Atlas (TCGA, https://www.cancer.gov/tcga) collates molecular data for more than 10,000 tumor and normal tissue samples across 33 different tumor types. The vast majority of the methylation data are from HM450, with some samples from HM27. All data from TCGA are available through the Genomic Data Commons (GDC) data portal^70^. Normalized methylation data, described in Gao et al.^71^, are available at https://portal.gdc.cancer.gov/, while the raw idat files can be retrieved through the GDC Legacy Archive (https://portal.gdc.cancer.gov/legacy-archive). Data from GDC can also be downloaded with tools such as TCGAbiolinks^72^. Moreover, data from individual TCGA studies are available at https://gdc.cancer.gov/about-data/publications, which also includes the complete datasets used in the PanCanAtlas Publications^73,74^ (https://gdc.cancer.gov/about-data/publications/pancanatlas). This web page provides all types of molecular data, including methylation data, and clinical data for all TCGA samples in simple text files.

The International Cancer Genome Consortium^75,76^ (ICGC) https://dcc.icgc.org/ is a resource similar to TCGA and also contains TCGA samples. Therapeutically Applicable Research to Generate Effective Treatments (TARGET) is similar to TCGA in the sense that it contains multiple types of molecular data, including methylation data, for several different tumor types. The data are available directly from https://ocg.cancer.gov/programs/target/data-matrix or through the GDC Data Portal.

Gene Expression Omnibus^77^ (GEO) and ArrayExpress^78^ are two public molecular data repositories with multiple methylation datasets available. The data can be accessed directly from their corresponding web pages through different application programming interfaces (APIs) and through some of the tools listed in Table 1.

Methylation data are also available from the Cancer Cell Line Encyclopedia^79^ (CCLE). Multiple types of molecular data, including reduced representation bisulfite sequencing (RRBS) methylation data, are available at https://portals.broadinstitute.org/ccle/data. The publication by Iorio et al.^80^ provides HM450 methylation data for 1028 CCLE cell lines and is available in GEO (GSE68379) or through ArrayExpress (E-MTAB-3610).

Compiled datasets from GEO, TCGA, and other sources are also available through different methylation databases. EWAS Data Hub^81^, DiseaseMeth^82,83^, and PubMeth^84^ are examples of such databases, but it should be noted that online tools may not always be updated and/or functional, while the source data from GEO, ArrayExpress, and TCGA are always available and up to date.

## Visualization of methylation data

Methylation data can be visualized similarly to other types of molecular data, such as gene expression data, e.g., with heatmaps, PCA plots and boxplots, but there are some unique features of methylation data that require some special considerations. The TCGA prostate adenocarcinoma (PRAD) methylation dataset will be used as an example dataset for different types of informative visualizations.

### Sample-to-sample density scatter plots

**Fig. 2:**
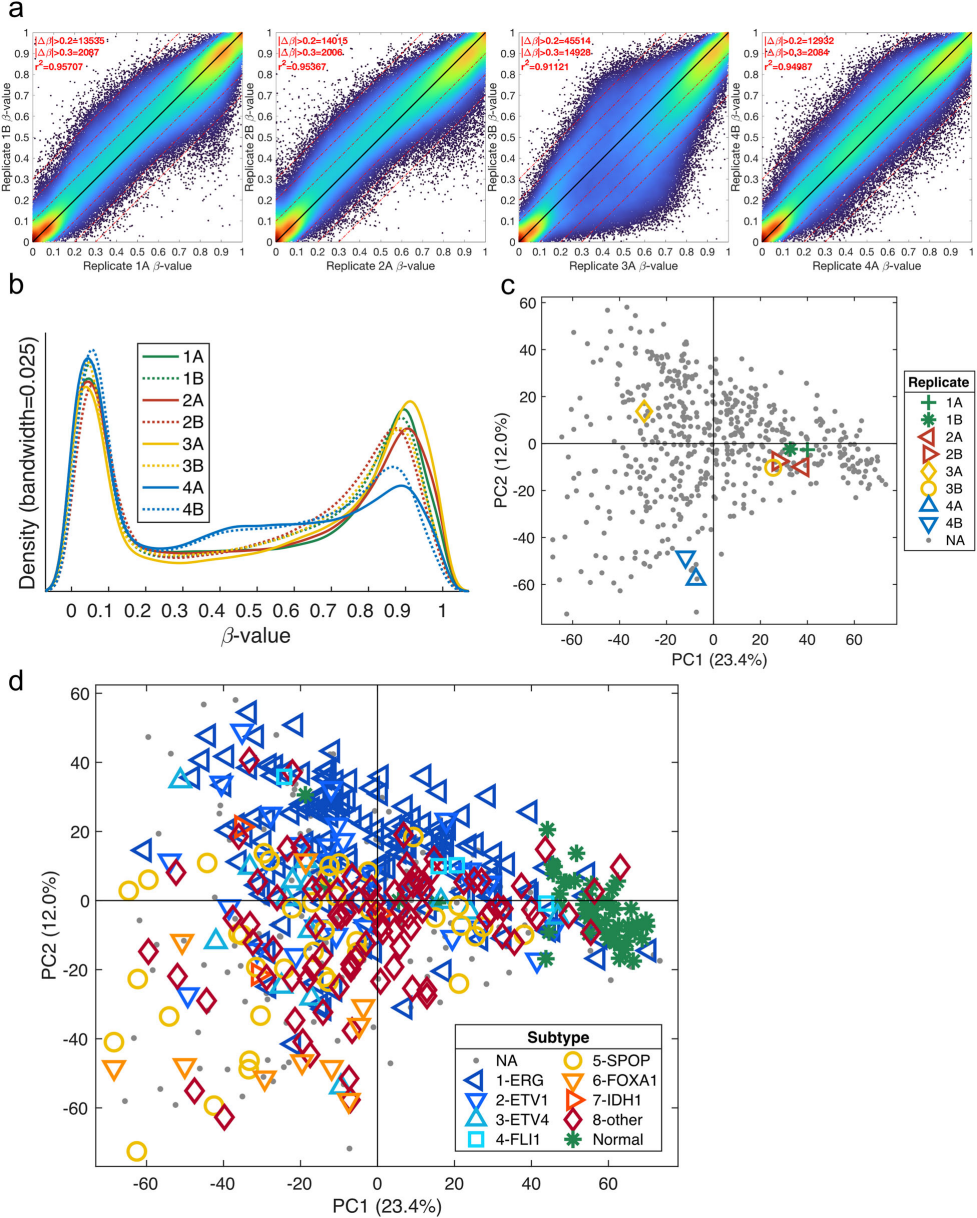
Visualization of methylation data. **a** Sample-to-sample density scatter plot for the four replicate pairs from the TCGA PRAD methylation dataset. The color indicates the density of the points, and the black line is the y=x line. The Pearson correlation coefficient is listed together with the numbers of probes with |Δβ| > 0.2 and |Δβ| > 0.3. **b** Histogram of beta-values for the four replicate pairs from the TCGA PRAD methylation dataset. **c** Scatter plot for the first two principal components, PC1 and PC2, from a PCA model using all the CpG probes and all the samples from the TCGA PRAD methylation dataset. The replicates are indicated by distinct shapes and colors. **d** Same PCA plot but with the colors and shapes based on the sample type and molecular subtype, respectively.

Replicates can be compared using sample-to-sample density scatter plots, as exemplified in Siegel et al.^85^. In Fig. 2a, four replicates from the TCGA PRAD study are compared by plotting all CpG probes in a scatter plot and assigning colors based on the density of each CpG probe. The density can quickly and easily be calculated using the algorithm described by Eilers and Goeman^86^. Even if there are several hundred thousand markers in the plot, the density clearly shows how similar each replicate pair is. Most CpG probes are located on or close to the black y=x line, indicating that the CpG probes have the same methylation value across the two replicates. The numbers of CpG probes that have a difference larger than 0.2 and 0.3 (|Δβ| > 0.2, (|Δβ| > 0.3) are listed in the top corner together with the correlation coefficient. It is clear from the density plots and the numbers that replicate 3 is different when compared to the other three replicate pairs.

### Beta-value histograms

Histograms of all the beta-values for a sample can be used to find low-quality samples. The beta-value for the eight replicate samples from the TCGA PRAD study is shown in Fig. 2b. A visual inspection of all samples can easily identify samples that do not have a clear bimodal distribution. It is clear from Fig. 2b that replicates 4A and 4B do not show the same bimodal distribution as the other samples, with a much lower right peak and a higher line in the middle. The distribution of beta-values is especially important for samples where there can be a mixed cell population and for FFPE samples where the DNA quality may be poor.

### PCA plots

Principal component analysis (PCA)^87^ score plots can be used to visualize all samples using all CpG probes to find groups, outlier samples, and batch effects. PCA is an unsupervised analysis method that displays the major trends in the data. Figure 2c shows the PCA score plot from a PCA model using the methylation data from the TCGA PRAD samples, including the replicates. The figure highlights the replicates and clearly confirms the findings from the two previous plots. Replicate pairs 1, 2, and 4 are similar to each other, while replicates 3A and 3B are located further from each other. The histogram in Fig. 2b indicates that replicates 4A and 4B are different, which is also confirmed in the PCA plot, since they are located far from the origin.

Figure 2d shows the same PCA score plot as Fig. 2c, but colors are instead assigned by biological subgroup, demonstrating separation based on biology. This plot indicates that the major changes in methylation arise from biological differences and not from batch effects or other technical artifacts. Using distinct colors and shapes improves the visual understanding of the PCA plot. The first principal component, PC1, explains 23.4% of the variation in the data and shows clear separation of the normal samples (green stars) and tumor samples. In other words, the methylation pattern in tumor samples is very different from the methylation pattern in normal samples. The PCA plot suggests that there is less variation in the normal samples (green stars) than in the different types of tumor samples. The second principal component, PC2, explains 12% of the variation and demonstrates separation of tumor samples with TMPRSS2 fusion (blue markers) from those without TMPRSS2 fusion (yellow-red markers). Thus, it becomes clear that the status of TMPRSS2 fusion results in methylation differences between different tumor types. This PCA plot also includes the ~150 samples (gray dots) not used in the TCGA PRAD publication due to RNA quality issues^88^. The PCA plot indicates that these samples are not different from the original 333 samples, since they are distributed across the whole area and are not separated as a unique group. It is recommended that probes with too many missing values (>50%) are removed before PCA, since they may become overly important to the PCA model. The nonlinear iterative partial least squares (NIPALS) algorithm can handle a moderate number of missing values, obviating the need to completely remove all probes with missing values. There are also available methods to impute missing values, which can be done before the PCA. CpG probes with a narrow range across all samples (e.g., 0.1) can also be removed before the PCA, since they are most likely describing nonbiological changes. It is also recommended to do the analysis on nonscaled data, i.e., without each variable scaled to unit variance. Scaling increases the importance of CpG probes with low variance, which have a lower signal-to-noise ratio. PCA can also be used to summarize the methylation level across multiple CpG probes and genes into one or a few variables^12^. When summarizing multiple variables using PCA, it is important to confirm that the PCA model describes the expected biology^89–91^.

### Gene-level visualization of methylation

**Fig. 3:**
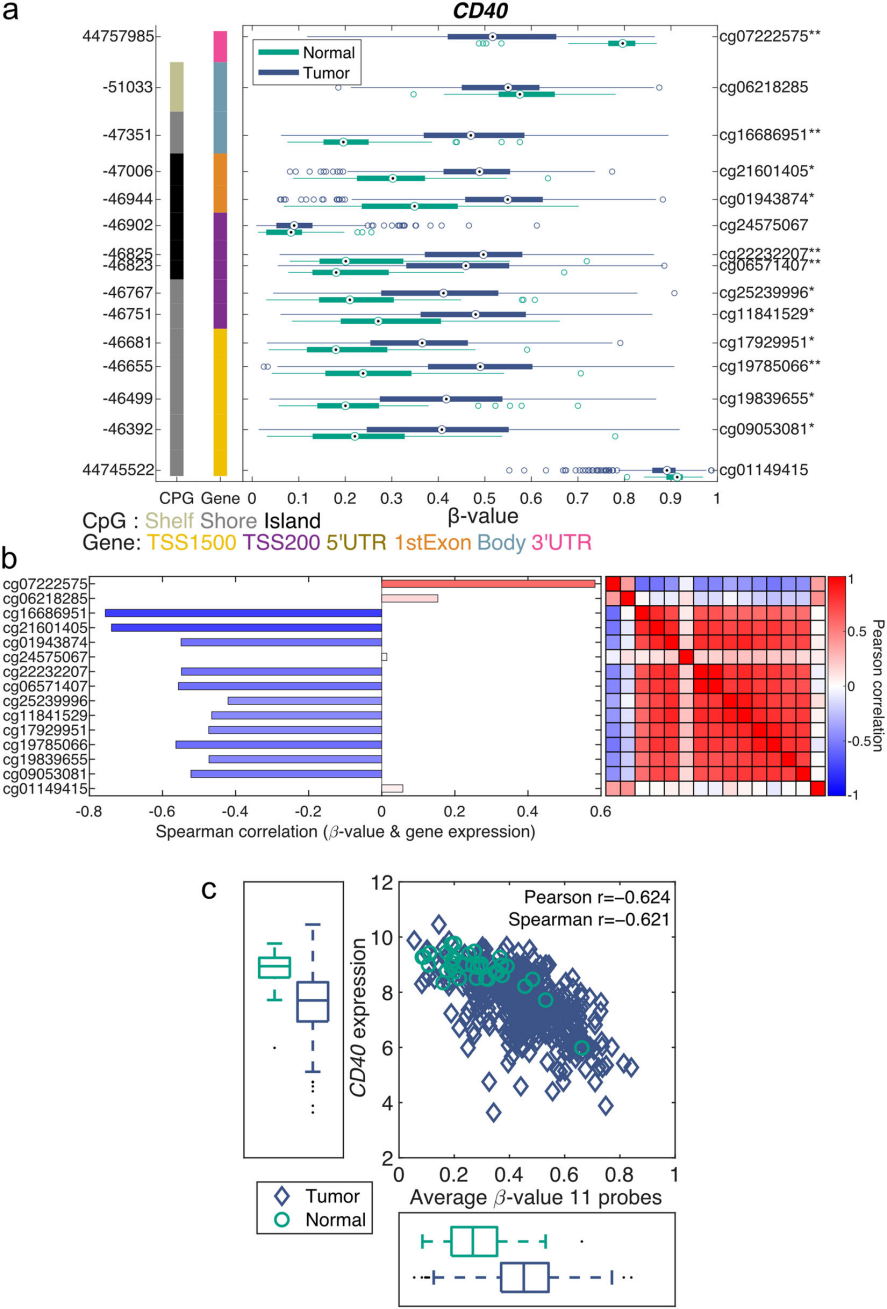
Gene-level visualization of methylation data. **a** Gene structure methylation (GSM) plot for *CD40* demonstrating the methylation of the 15 CpG probes across the tumor and normal samples from the TCGA PRAD dataset. The *x*-axis shows the beta-values, which are shown in boxplots for each CpG probe and group, with normal samples shown in green and tumor samples in blue. The left *y*-axis displays the genomic position, while the right *y*-axis displays the probe id. The leftmost vertical column indicates CpG islands, and the right vertical column indicates the gene structure location. *q < 0.05 and |Δβ| > 0.1, **q < 0.01 & |Δβ| > 0.2. **b** The bar plot shows the Pearson correlation coefficient between each *CD40* CpG probe and the gene expression level for the TCGA PRAD samples. The heatmap demonstrates the Pearson correlation of the methylation level between each CpG-probe pair. **c** The *CD40* RNAseq gene expression level vs. the average methylation level for the 11 selected *CD40* CpG probes using TCGA PRAD samples. Normal samples are shown as green circles, and tumors are shown as blue diamonds.

Visualizing methylation data for a gene has several challenges. Frequently, there are several CpG probes available for each gene, and the methylation pattern for each CpG probe can be quite different, which makes it problematic to select the CpG probes to include when representing the gene. Using rules such as the position in the gene body (TSS1500, TSS200, 5’UTR, 1st exon, body, or 3’UTR) is not an optimal option, since a CpG probe can be located in different gene regions when the gene has multiple transcripts. Representing the gene by a single CpG probe is not ideal either, since this approach does not use all available data and is more sensitive to missing values and noise. There is also no guarantee that CpG probes within the same gene body region have similar methylation patterns. Displaying the methylation levels across all CpG probes for a gene as boxplots across multiple groups—for example, normal vs. tumor—provides valuable information that can be used when selecting CpG probes. One example of such a plot is the gene structure methylation (GSM) plot^92^, shown in Fig. 3a. Figure 3a shows the methylation levels of the 15 CpG probes for *CD40* in the TCGA PRAD dataset. The methylation level of each CpG probe is shown along the *x*-axis (ranging from 0 to 1) and is represented by a boxplot for each group, with normal samples shown in green and tumor samples in blue. The genomic location is displayed on the left y-axis, while the right *y*-axis shows the probe ID with a star (*) indicating a significant difference between the normal and tumor samples. The distance between each probe on the *y*-axis is logarithmically related to the number of base pairs between the two probes. The leftmost column indicates the presence of CpG islands, and the right column indicates the CpG probes’ positions in the gene structure.

Twelve of the fifteen *CD40* CpG probes show a significant difference in methylation between the normal and tumor samples. Eleven of the fifteen *CD40* CpG probes demonstrate hypermethylation in the tumor samples compared to the normal samples, while one CpG probe, cg07222575, shows the opposite trend, with a higher degree of methylation in normal samples. Two of the CpG probes, cg24575067 and cg01149415, demonstrate less variation, with cg24575067 showing a low degree of methylation and cg01149415 showing a high degree of methylation in both normal and tumor samples. This finding clearly exemplifies that CpG probes can show very different methylation patterns for the same gene.

This observation is further confirmed in the correlation heatmap in Fig. 3b, which illustrates the correlation between each probe. The top two probes, cg07222575 and cg06218285, are correlated to each other and to the bottom CpG probe, cg01149415, while showing a negative correlation with all the other CpG probes. Eleven probes, ranging from cg16686951 to cg09053081 and excluding cg4575067, are highly correlated with each other and show a significantly higher methylation level in tumor samples than in normal samples (Fig. 3a). This pattern indicates that the expression of *CD40* is suppressed in tumors compared to normal tissues. The bar plot in Fig. 3b clearly demonstrates that these eleven CpG probes show a negative correlation with the gene expression level of *CD40*. These three figures clearly demonstrate that using the average of these eleven CpG probes provides a good representation of the *CD40* methylation level.

The average methylation level of the 11 selected *CD40* CpG probes is clearly negatively correlated with the *CD40* gene expression level identified by RNAseq, as shown in Fig. 3c. This finding indicates that *CD40* is at least partially regulated by methylation in prostate cancer.

## Online visualization of methylation data

The figures and graphs described in the previous section can be generated in many programming languages, such as R, MATLAB, Python, and Julia, and through some of the pipelines described in Table 1. Methylation data can also be analyzed and visualized using online tools with preloaded data from TCGA, GEO, and other sources. These tools are great resources for researchers with limited bioinformatics or programming expertise.

DNMIVD (DNA Methylation Interactive Visualization Database) is a web-based tool with many features^93^. DNMIVD uses TCGA methylation data, RNAseq gene expression data, and survival data together with the cancer hallmark pathways from Zhang et al.^94^. Both CpG-level and gene-level information are available together with survival information and various quantitative trait loci (QTL) results.

The SMART App is an interactive web application through which TCGA data are also available^95^. Normal vs. tumor boxplots are provided for specific CpG probes but can also be aggregated for selected CpG probes. Similar boxplots can also be generated to visualize the stage and mutational status. In addition, correlations between methylation and gene expression levels, as well as survival curves, are available.

Wanderer^96^ provides CpG-probe-level visualization of the methylation status for TCGA data, including both normal and tumor samples. CpG-probe correlation plots for RNAseq data are also available. All results for normal and tumor samples are separated. The data can easily be downloaded.

MEXPRESS^97,98^ provides a slightly different type of visualization of TCGA methylation data, where many types of clinical data are overlaid with clinical annotations.

MethSurv^99^ is focused on the survival analysis of patients with 25 types of cancer from TCGA. The analysis is CpG probe specific only, and no gene expression data are available. There are options to create both heatmaps and PCA plots.

## Summary

Methylation is one of the main epigenetic mechanisms controlling gene transcription and is thus increasingly recognized as one of the major mechanisms of cancer immune escape. As cancer cells evade the immune system via dysregulated methylation of immune pathway-related genes during carcinogenesis, these alterations evolve into resistance mechanisms against the current immunotherapeutic agents. Epigenetic dysregulation of immune-related pathways in cancers may be mediated by natural selection and clonal expansion of cancer cells that display the immune-evasive phenotype acquired in response to immune surveillance. This evolutionary concept implies the crucial role of tumor-immune crosstalk throughout carcinogenesis and underscores the need for in-depth characterization of the role of methylation in modulating antitumor immune responses, which may reveal actionable targets to overcome immune evasion. There exists a vast amount of methylation data that are ready to be analyzed using well-established bioinformatics pipelines. The results can be visualized in informative figures that clearly show the epigenetic changes in tumor samples.
